# Achromatic nested Kirkpatrick–Baez mirror optics for hard X-ray nanofocusing

**DOI:** 10.1107/S0909049511010995

**Published:** 2011-05-12

**Authors:** Wenjun Liu, Gene E. Ice, Lahsen Assoufid, Chian Liu, Bing Shi, Ruben Khachatryan, Jun Qian, Paul Zschack, Jonathan Z. Tischler, J.-Y. Choi

**Affiliations:** a Advanced Photon Source, Argonne National Laboratory, 9700 South Cass Avenue, Argonne, IL 60439, USA; b Oak Ridge National Laboratory, Oak Ridge, TN 37831, USA; c Pohang Accelerator Laboratory, Pohang, Republic of Korea

**Keywords:** hard X-ray nanofocusing, achromatic, nested Kirkpatrick–Baez, Montel

## Abstract

The first test of nanoscale-focusing Kirkpatrick–Baez (KB) mirrors in the nested (or Montel) configuration used at a hard X-ray synchrotron beamline is reported. The two mirrors are both 40 mm long and coated with Pt to produce a focal length of 60 mm at 3 mrad incident angle, and collect up to a 120 µm by 120 µm incident X-ray beam with maximum angular acceptance of 2 mrad and a broad bandwidth of energies up to 30 keV. In an initial test a focal spot of about 150 nm in both horizontal and vertical directions was achieved with either polychromatic or monochromatic beam. The nested mirror geometry, with two mirrors mounted side-by-side and perpendicular to each other, is significantly more compact and provides higher demagnification than the traditional sequential KB mirror arrangement. Ultimately, nested mirrors can focus larger divergence to improve the diffraction limit of achromatic optics. A major challenge with the fabrication of the required mirrors is the need for near-perfect mirror surfaces near the edge of at least one of the mirrors. Special polishing procedures and surface profile coating were used to preserve the mirror surface quality at the reflecting edge. Further developments aimed at achieving diffraction-limited focusing below 50 nm are underway.

## Abstract 2

A nested Kirkpatrick–Baez mirror pair has been designed, fabricated and tested for achromatic nanofocusing synchrotron hard X-rays. The prototype system achieved a FWHM focal spot of about 150 nm in both horizontal and vertical directions.

## 1. Introduction

**Figure 1:**
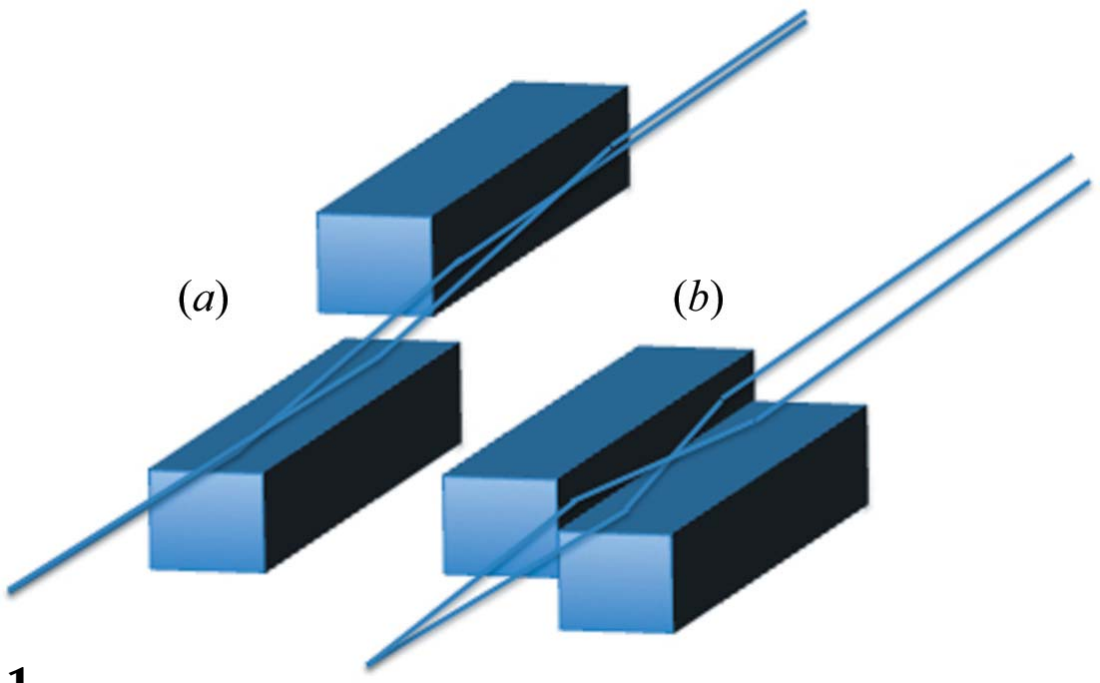
(*a*) Schematic of a standard (sequential) KB mirror arrangement. (*b*) Schematic of a nested (Montel) mirror pair.

Although synchrotron micro/nanofocusing mirror optics are currently dominated by traditional Kirkpatrick–Baez (KB) mirrors (Kirkpatrick & Baez, 1948 ▶), nested KB or Montel mirror optics (Montel, 1957 ▶) are a desirable goal because of their compact design with stronger demagnification and the ability to collect larger divergences. Recent papers have described their advantages for neutron microfocusing (Ice *et al.*, 2009*a* ▶) and have described ray-tracing programs for modelling synchrotron applications (Honnicke *et al.*, 2010 ▶). To understand the advantages of nested KB optics compared with traditional KB optics we compare the two designs as illustrated in Fig. 1 ▶. With traditional KB optics (Fig. 1*a* ▶), X-rays are focused by sequential elliptical surfaces. This approach allows for the fabrication of ultra-precise mirror surfaces and has been used to create the smallest doubly and singly focused beams to date (Mimura *et al.*, 2007 ▶, 2010 ▶). With nested KB optics, however, the two elliptical mirrors are positioned side-by-side and perpendicular to each other (Fig. 1*b* ▶). The X-rays strike both surfaces at the same time. This geometry has four important advantages for high-precision focusing: (i) the mirror system is more compact, which allows greater working distance to the sample; (ii) the focal distance of the mirrors is much shorter than for the primary mirror of a comparable sequential KB system, which creates a greater geometrical demagnification of the source and reduces the effect of figure errors (in one direction); (iii) the mirrors can be easily aligned to be orthogonal which is critical for best focusing (Matsuyama *et al.*, 2005 ▶); and (iv) the divergence that can be collected is larger which allows for greater flux and/or a lower diffraction limit (Ice, 2008 ▶; Ice *et al.*, 2009*b* ▶).

The theoretical limits of sequential KB and Montel optics were quantified in a paper (Ice *et al.*, 2009*b* ▶) that compared the performance of both mirror systems as a function of the ratio, *n* = *L*/*C*, of the final mirror length, *L*, to the clearance from the end of the last optical element, *C*. Assuming the mirror systems collect equal divergences in both directions, the length of Montel optics, *nC*, compares with a similar KB system with a length (2*n* + *n* ^2^)*C*. For mirror systems like the prototype described in this paper, where *L*/*C* = *n* = 1, the difference in length of the system is a factor of at least 3. If *n* = 2 or even 3, which can significantly improve the diffraction limit, then the difference in system lengths between KB and Montel optics becomes a factor of 8 or 15, respectively.

In recent years many efforts have been made to use multilayer mirrors to increase the numerical aperture for lowering the diffraction limit (Mimura *et al.*, 2010 ▶; Morawe & Osterhoff, 2009 ▶; Kang *et al.*, 2006 ▶). However, multilayer mirror optics typically have a restricted energy bandwidth. To preserve achromatic focusing performance, total-external-reflection X-ray mirrors are still essential for applications such as diffraction experiments and extended X-ray absorption fine-structure measurements.

## 2. Mirror system design and fabrication

### 2.1. Optical design

**Figure 2:**
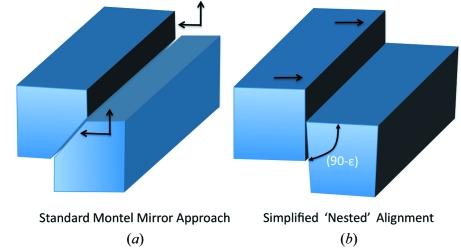
(*a*) Montel mirror pair formed by cutting a prefigured mirror at 45° to the surface. (*b*) Montel mirror pair formed by shaping the edge of one mirror and then nesting it into the curvature of the companion mirror.

In a Montel system the mirror surfaces must come together at the mirror plane that divides the two reflecting surfaces. Two methods can be used to produce mirrors assembled into a Montel pair. One is to cut a prefigured mirror into two parts and grind the edges at a 45° angle to the surface, as shown in Fig. 2(*a*) ▶. This configuration allows a perfect fit of two reflecting mirrors with no gap at the corner. However, with mirrors of this design the edges of both mirrors must be used, *i.e.* both mirrors will need high-quality edge polishing, and also the mirrors must be aligned along two axes at both ends of the mirror pair. Another way to produce a Montel pair is by cutting the edge of one mirror at slightly less than 90°, as shown in Fig. 2(*b*) ▶. This approach requires that the edge not only has a right angle to the surface, but that it also has an elliptical profile to ‘nest’ against the companion mirror to make an almost perfect fit. The advantage of this approach is that only the edge of one mirror must be used, and the alignment is primarily one-dimensional at each end of the mirror pair. This 90° nesting approach was adopted for our prototype device.

**Figure 3:**
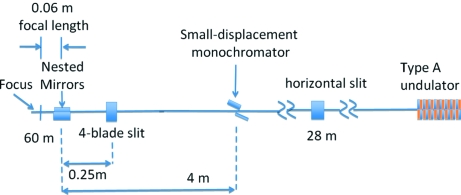
Schematic of the beamline layout of 34-ID indicating the key optical elements and their locations.

The prototype Montel system has been designed for hard X-ray nanofocusing at the 34-ID-E station of the Advanced Photon Source (APS). Fig. 3 ▶ schematically illustrates the beamline layout of 34-ID and the nanofocusing set-up. The experimental station is located about 60 m from the source. A horizontal slit at 28 m was placed to control the total power in the beam and to reduce the horizontal source size down to <100 µm; thus it also acts as a new effective object. In the vertical plane the APS type-A undulator source, with FWHM of about 40 µm, serves directly as the object (Liu *et al.*, 2005 ▶).

The two elliptical mirrors are both 40 mm long and coated with Pt to produce an identical focal length of 60 mm at 3 mrad incident angles. They can accept up to a 120 µm by 120 µm incident X-ray beam with a broad bandwidth of energies from 7 to 30 keV. The mirror optics have demagnifications of about 530:1 horizontally and 1000:1 vertically. Table 1 ▶ lists all the key optical parameters of the prototype nested-mirror nanofocusing system.

### 2.2. Mirror edge polishing, surface profile coating and metrology

**Figure 4:**
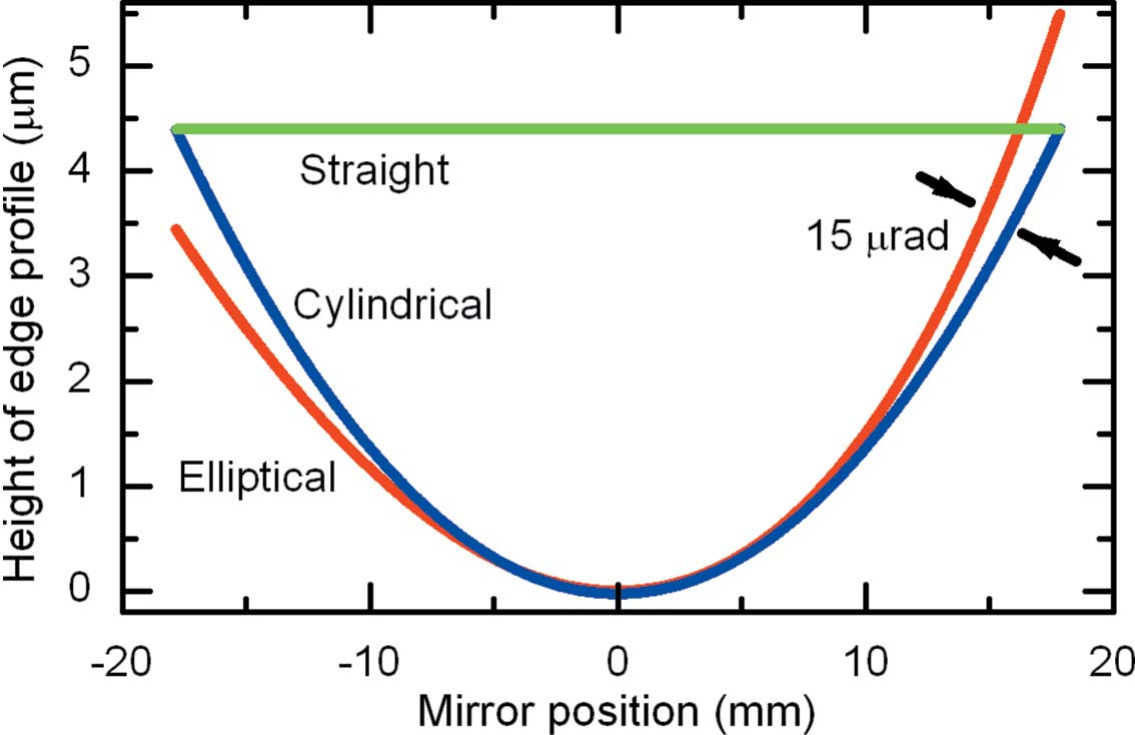
The edge of the nested mirror must be shaped to an ellipse to avoid lost rays at the corner. However, even a straight-line approximation is not too bad. For example, for the prototype mirrors the maximum missing mirror surface is about 5 µm wide and the total area lost is about 1.3 × 10^5^ µm^2^. This will cost about 6.5% for a 100 µm × 100 µm incident beam. If the edge is profiled to a simple cylinder and tilted slightly (∼15 µrad), the missing mirror area is reduced to 0.5% of the total mirror surface used.

The main challenge of nested mirror fabrication and assembly is to preserve the mirror surface quality at the reflecting edge and to shape the mirror edge so that it nests against the elliptical surface of the partner mirror. When the edge of the mirror that is placed against the elliptical surface of its companion mirror is a straight line, intensity is lost from the doubly focused beam if either first or second reflections occur at the gap between the mirrors. This sacrifices a small (<7%) portion of the mirror edge reflecting X-rays. However, a simple cylindrical edge can be used to dramatically reduce the missing portion of the mirror to below 0.5%, shown in Fig. 4 ▶. In order to make a best fit of a cylindrical edge to the elliptical surface profile of the companion mirror the nesting mirror can be in-plane tilted at ∼15 µrad. This small yaw adjustment will have a negligible consequence on focusing (Matsuyama *et al.*, 2005 ▶).

To simplify the polishing for the prototype test, the edge of the nesting mirror was polished to an approximately straight line. Two identical flat-mirror substrates with dimensions of 40 mm (L) × 9 mm (W) × 20 mm (H) were chosen for producing a nested mirror pair. One of the mirrors was side-polished to have a <1° chamfer. The edge of slightly less than 90° made it possible to nest the mirror surfaces in close contact. The quality of the mirror edge after polishing is expected to have a roughness of about 0.1 nm r.m.s. and figure error of <1 nm peak-to-valley. However, chipping and micro-cracking at the edge are observed. This will be discussed later.

A profile-coating technique was used to convert inexpensive flat or spherical Si substrates into precise elliptical mirror surfaces (Liu *et al.*, 2002 ▶; Ice *et al.*, 2000 ▶). The technique utilizes a contoured aperture mask in a DC magnetron sputtering system with linear motion to coat a predetermined profile onto mirror substrates. The shape of the contour is calculated according to the desired elliptical profile of an ideal final mirror and from the measured shape of the original substrate surface. Platinum (Shi *et al.*, 2011 ▶) has been successfully used as coating materials. Very precise elliptical KB mirrors with sub-nanometre r.m.s. height errors have been obtained with one primary profile-coating followed by one or two corrective profile-coating procedures.

**Figure 5:**
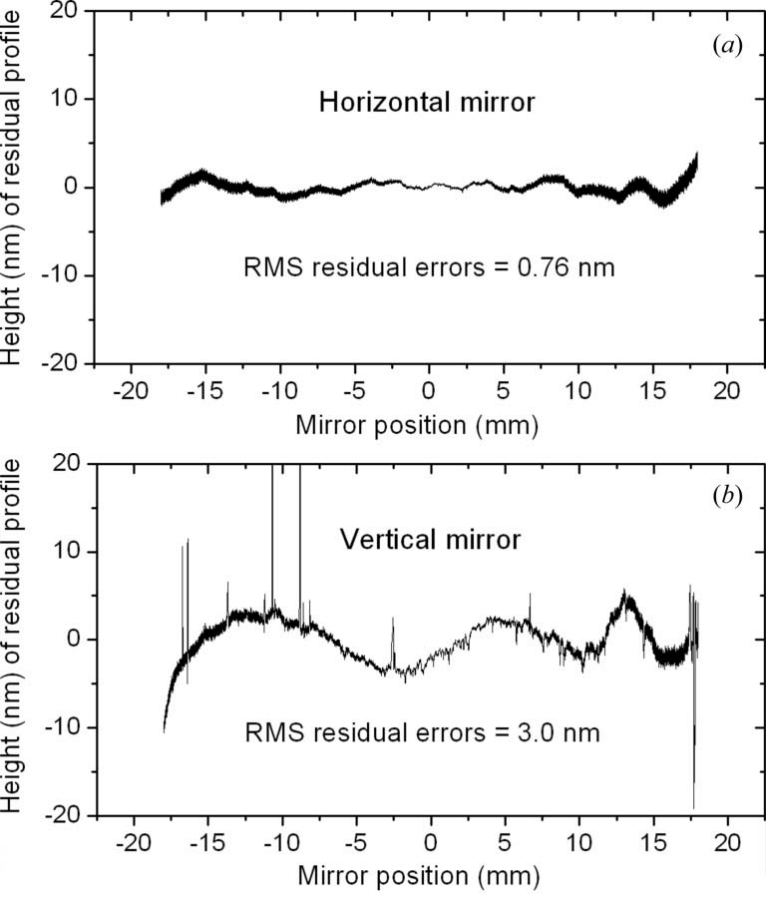
Metrology on both horizontal and vertical reflecting mirrors. (*a*) Horizontal mirror. (*b*) Side-polished vertical mirror near the edge. The spikes are from small chips at the edge.

All the nested KB mirrors were profile-coated with platinum. Metrology measurements were carried out using a stitching interferometer (Assoufid *et al.*, 2007 ▶). Fig. 5(*a*) ▶ shows profile results of the horizontal focusing mirror which was not edge-polished, and Fig. 5(*b*) ▶ shows the profile at the edge of the vertical focusing mirror after cutting, polishing and coating. A very sharp edge, within a few micrometres of the design, was obtained. The metrology result indicates that 0.76 nm r.m.s. height-error-accuracy remains in the horizontal mirror. However, for the vertically deflecting mirror surface after side-polishing, the r.m.s. of the profile is about 3.0 nm. The increased r.m.s. values are due to chips at the edge, shown in the metrology measurement as sharp spikes.

### 2.3. Mirror assembly

**Figure 6:**
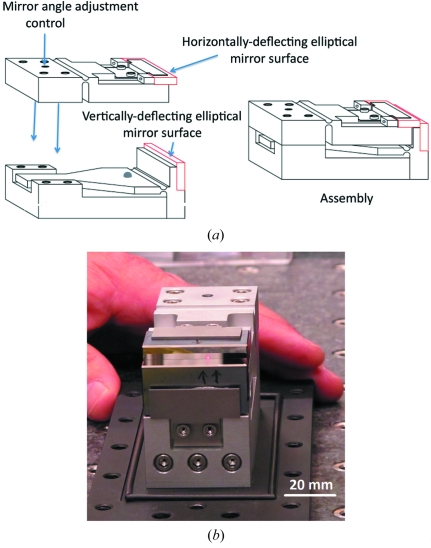
(*a*) Schematic of the Montel mirror pair assembly. The mirror position and orthogonality were preset on a flexure-based fixture with no motorized parts. (*b*) Picture of the assembled mirror pair.

**Figure 7:**
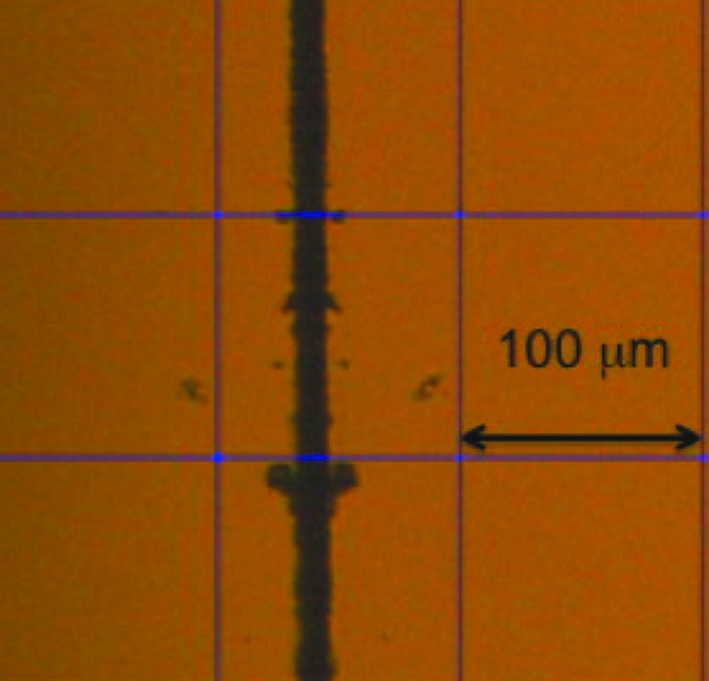
Optical micrograph showing the gap between the two mirror surfaces. The measured gap was ∼7 µm near the mirror centre whereas the ideal gap should have been less than 5 µm.

The mirrors were mounted on a small specially designed fixture that allowed the horizontally deflecting mirror to be nested tight against the vertically deflecting mirror and rotated to make the two mirrors precisely orthogonal to each other. A schematic of the fixture is shown in Fig. 6(*a*) ▶, and Fig. 6(*b*) ▶ shows the assembled mirror pair. The mirrors were brought together manually, by sliding the horizontal mirror up against the vertical mirror. An optical micrograph of the assembled corner is shown in Fig. 7 ▶. The apparent gap between the mirrors as seen in the microscope is about twice as large as the actual image owing to the optical image of the gap reflected off the vertically deflecting mirror surface. The actual gap was estimated to be about 8 µm, whereas with ideal positioning the gap should have been less than 5 µm. The orthogonality was checked by monitoring a laser beam reflected from the corner where the two mirrors come together. If the two mirrors are not precisely orthogonal, the beam paths will reflect through paths that differ by 4δ, where δ is the angular deviation from 90°. As a result there are typically two spots reflected by the alternative paths at mirrors corner. By adjusting the tilt, these spots are brought together and the mirror orthogonality is easily set to 100 µrad or less. This level of orthogonality is adequate for focusing 120 µm beams to a spot of tens of nanometres.

## 3. X-ray testing and mirror focusing performance

The prototype hard X-ray nanofocusing system based on nested KB mirror optics has been installed and tested at station 34-ID-E at the APS. The station is dedicated to three-dimensional Laue diffraction microscopy for materials science applications (Liu *et al.*, 2004 ▶), and includes a six ton (∼5400 kg) granite optical table for testing new optical designs and for the development of a diffraction nanoprobe. A removable small-displacement Si (111) double-crystal monochromator, located 56 m from the source, allows rapid X-ray beam change between monochromatic mode and polychromatic mode. The mirror assembly was mounted on a Newport six-axis hexapod stage for positioning and alignment of mirrors in both horizontal and vertical directions in the incident X-ray beam. A JJ X-ray four-blade beam-defining slit in front of the focusing mirrors was used to limit the incident beam acceptance.

To measure the focal spot, a series of thin Au film stripes are scanned across the beam at a glancing angle of 4 mrad. Each stripe is equivalent to a ∼20 nm-wide pseudo-slit or reflector. Either Au fluorescence or the reflected intensity by film was collected (Liu *et al.*, 2005 ▶). The patterned multiple nanoslits/reflectors can profile the beam at 200 µm increments along the beam axis, so that the focal point can be quickly located and precisely measured. At the exit of the mirror enclosure an L-shaped beam stop was placed to allow only X-rays reflected from both mirrors to pass.

**Figure 8:**
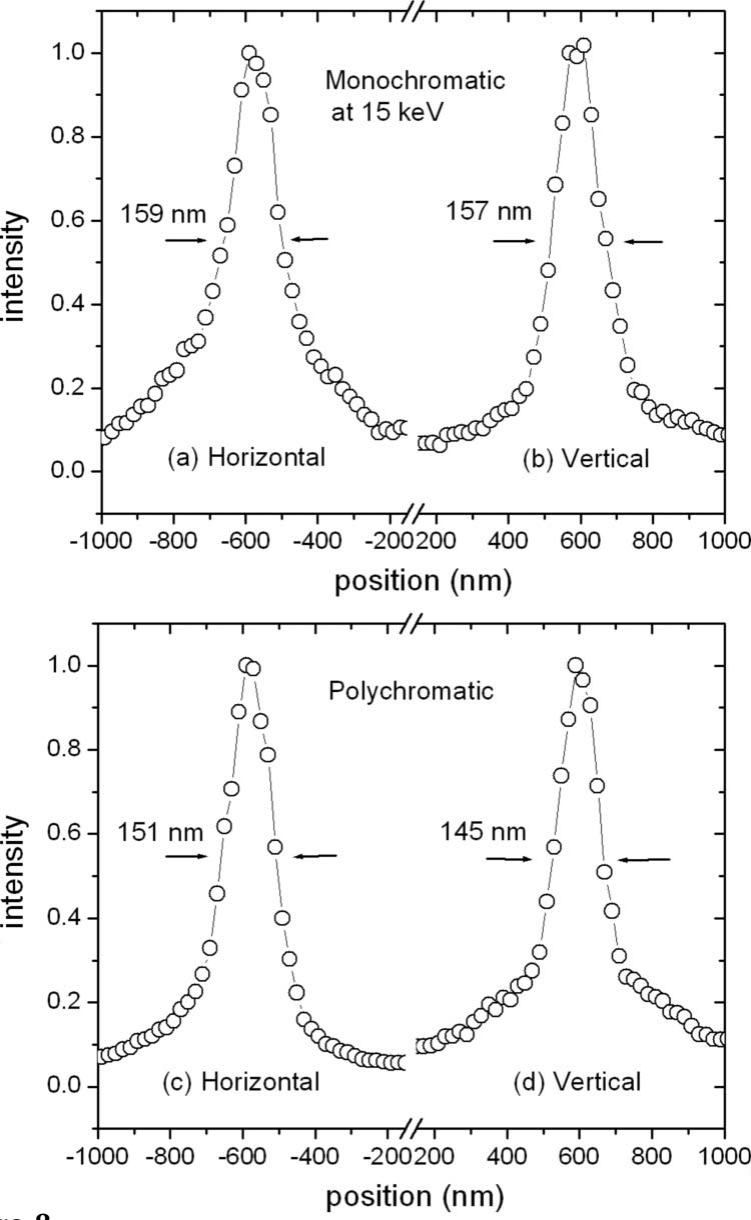
Horizontal and vertical measurements of doubly focused spots, with a monochromatic X-ray beam at 15 keV (*a*, *b*), and with a polychromatic X-ray beam (*c*, *d*).

The nested mirrors can collect up to a 120 µm by 120 µm incident X-ray beam at 3 mrad incident angles. In the actual measurements, 100 µm × 100 µm and 50 µm × 50 µm beams were used with small adjustments in the mirror positions to search for the best part of the mirror surfaces. The mirror angles were adjusted to optimize the focal spot size. As shown in Figs. 8(*a*) and 8(*b*) ▶ a doubly focused spot of ∼159 nm horizontal × 157 nm vertical was achieved with monochromatic beam at 15 keV. Similar polychromatic measurements were also made, and a slightly smaller spot size of 151 nm horizontal × 145 nm vertical was obtained (Figs. 8*c* and 8*d* ▶). This indicates that there may be some focal blurring introduced by the monochromator.

The transmission efficiency of the optics was checked by measuring the total flux in an ion chamber with or without the focusing mirrors. Measurements were performed at 11 keV to avoid the Pt *L*-absorption edges. Theoretically, one mirror should have a reflectivity of 94%, while two mirrors should have a combined reflectivity of 89%. The measured reflectivity was 92% from the horizontal focusing mirror, which was close to theory. However, when the edge-polished vertical mirror was brought together with the horizontal mirror, the overall reflectivity of the nested mirror system became 45%. This indicates significant losses of flux near the edge of the vertical mirror. With an ideal straight edge, losses are only expected to be ∼7%. As seen in the optical micrograph of the assembled mirror pair (Fig. 7 ▶), the measured gap could explain up to a ∼15% loss of flux. The additional losses are believed to be due to chipping of the edge. Interestingly, the focal size of the vertical mirror was about the same as that of the horizontal mirror. There were no significant tails observed at the focal plane, which means that the vertical mirror slope was not significantly affected by the edge chipping.

## 4. Conclusion

The nested KB prototype demonstrates the potential of Montel focusing optics to reduce the focal spot size of achromatic optics. In principle, perfect optics of this kind can reach diffraction-limited two-dimensional focusing, and can improve the geometrical demagnification compared with traditional sequential KB optics. The current prototype is limited by several factors including mirror imperfection, beamline geometrical demagnification, vibrations of the optical system, and thermal beam instabilities. At beamline 34-ID-E of the APS the geometrical demagnifications, as shown in Table 1 ▶, allow for an ideal focal spot of ∼40 nm in both the vertical and horizontal directions. Mechanical vibration and temperature drift control were measured to be better than 30 nm. The mirror orthogonality of less than 100 µrad is adequate for focusing 120 µm beams to spot sizes of tens of nanometres. Therefore, focus blurring was mainly due to the mirror imperfection of our prototype optical system. Improved mirror fabrication with higher performance is needed. New polishing procedures have since been developed to eliminate virtually all the edge chipping. The focusing efficiency is expected to significantly increase by side-polishing the mirror to make a cylindrical edge. Better mirror control using a high-stiffness tip-tilting stage system with nanoradian-level multidimensional positioning resolution is also under development. Ultimately, KB mirrors in the Montel arrangement are important for non-dispersive nanofocusing of hard X-rays over a wide bandwidth. Because of its significant compactness and higher demagnification compared with a traditional sequential KB mirror arrangement, it is particularly appealing to use the nested geometry in conventional (∼60 m) synchrotron beamlines, which usually do not have sufficient geometrical demagnification to achieve a sub-100 nm focal spot with a practical working distance.

## Figures and Tables

**Table 1:** Optical parameters of the nested-mirror focusing system

	Mirror length (mm)	Focal length (mm)	Geometrical demagnification	Mirror glancing angle (mrad)	Maximum beam acceptance (µm)	Maximum angular acceptance (mrad)
Vertical mirror	40	60	1000	3.0	120	2.0
Horizontal mirror	40	60	530	3.0	120	2.0
